# Neurocognitive impairment and substance use in adult survivors of childhood cancer: a cross-sectional analysis from the Childhood Cancer Survivor Study

**DOI:** 10.1016/j.eclinm.2026.103924

**Published:** 2026-04-29

**Authors:** Rachel Webster, Eleanor O. Chambers, Weiyu Qiu, Rikeenkumar Dhaduk, Lei Wang, Yutaka Yasui, Yan Yuan, Paul C. Nathan, Wendy Leisenring, Gregory T. Armstrong, Rebecca Howell, Tilman Schulte, Matthew Yalch, Matthew Cordova, Kevin R. Krull, Tara M. Brinkman, Kim Edelstein

**Affiliations:** a Department of Psychology and Biobehavioral Sciences, St. Jude Children’s Research Hospital, Memphis, TN, USA; b Psychology Department, Palo Alto University, Palo Alto, CA, USA; c School of Public Health, University of Alberta, Edmonton, Alberta, T6G1C9, Canada; d Department of Biostatistics, St. Jude Children’s Research Hospital, Memphis, TN, USA; e Department of Epidemiology and Cancer Control, St. Jude Children’s Research Hospital, Memphis, TN, USA; f Department of Pediatrics, The Hospital for Sick Children, University of Toronto, Toronto, Canada; g Clinical and Public Health Sciences Divisions, Fred Hutchinson Cancer Center, Seattle, WA, USA; h Department of Radiation Physics, MD Anderson Cancer Center, Houston, TX, USA; i Princess Margaret Cancer Centre, University Health Network, Toronto, Ontario, Canada

**Keywords:** Childhood cancer survivors, Substance use, Neurocognitive impairment, Psychological distress, Pain

## Abstract

**Background:**

Adult survivors of childhood cancer are at risk for neurocognitive impairment, emotional distress, and chronic pain, factors independently associated with substance use. However, interrelationships among these factors and their association with substance use remain poorly understood.

**Methods:**

Participants from the Childhood Cancer Survivor Study (CCSS) who reported symptoms of neurocognitive problems, distress, pain, and substance use were included. Data for these cross-sectional analyses were drawn from CCSS follow up questionnaires administered between 2003 and 2007. Polytomous regressions examined associations between neurocognitive impairment and substance use (alcohol: occasional, risky, heavy; smoking: current) and whether these associations were moderated by psychosomatic symptoms. The CCSS cohort study is registered with ClinicalTrials.gov, NCT01120353.

**Findings:**

11,151 participants were included (53.2% female; mean age 31.4 years, SD 7.5). Survivors reported risky (40.9%; n = 4059/9894), or heavy (11%; n = 1096/9894) alcohol use and more than 25% reported previous (14.6%; n = 1482/10,182) or current (13.7%; n = 1395/10,182) cigarette use. Impaired emotional regulation was associated with smoking (odds ratio 1.81, 95% CI 1.53–2.14) and risky drinking (1.59, 1.25–2.03). Impaired emotion regulation with somatisation was associated with decreased occasional (0.54, 0.0–0.95) and heavy drinking (0.54, 0.0–0.95). Organisation impairment with somatisation was associated with decreased heavy drinking (0.34, 0.15–0.73), whereas pain with organisation impairment was associated with increased occasional (1.81, 1.03–3.19) and risky drinking (2.18, 1.24–3.85). Memory impairment was associated with risky (1.38, 1.10–1.73) and occasional drinking (1.37, 1.09–1.71).

**Interpretation:**

Neurocognitive impairment was associated with substance use and modified by psychosomatic symptoms. Findings support integrated screening to inform targeted interventions.

**Funding:**

National Cancer Institute, Princess Margaret Cancer Centre Foundation, Ontario Ministry of Health and Long-Term Care, National Cancer Institute Cancer Center, and American Lebanese Syrian Associated Charities.

**Research in context**

**Evidence before this study**

We searched PubMed, Google Scholar, and reviewed citations of relevant articles from database inception through early 2025 using the following terms: childhood cancer survivors, pediatric cancer survivors, neurocognitive impairment, substance use, alcohol use, risky alcohol use, heavy alcohol use, cigarette use, psychological distress, depression, somatisation, anxiety, pain, pain symptoms. Previous research has established that neurocognitive impairment, emotional distress, and chronic pain are independently associated with substance use in adult survivors of childhood cancer; however, the associations among these factors and their combined influence on substance use in childhood cancer survivors remains poorly understood. Understanding how neurocognitive deficits interact with distress and pain to influence substance use may identify critical targets for intervention.

**Added value of this study**

This study examined domain-specific neurocognitive impairments (task efficiency, emotional regulation, organisation, memory) and their interactions with psychological distress and pain in relation to substance use among a large, well-characterised cohort of adult childhood cancer survivors (N = 11,151). Impairments in emotional regulation and memory were associated with increased odds of substance use. Moderation analyses revealed that co-occurring symptoms, such as depression, somatisation, and pain, altered these associations, sometimes reversing risk patterns. These findings underscore the complexity of behavioural risk in survivorship.

**Implications of all the available evidence**

These findings support moving beyond universal survivorship screening toward risk-stratified approaches that integrate domain-specific neurocognitive vulnerabilities with co-occurring distress and pain symptoms. Survivors with neurocognitive impairments, particularly when accompanied by pain or somatic distress, may warrant enhanced monitoring and targeted preventive interventions rather than reliance on general health behaviour counselling alone.

## Introduction

Young adult survivors of childhood cancer face elevated risks for treatment-related chronic health conditions^1^, ^2^, ^3^ Despite these risks, many engage in health-compromising behaviours with tobacco use (10–29%) and risky alcohol consumption (∼77%) prevalent among cancer survivors.^4^, ^5^, ^6^ These behaviours (hereafter referred to as “substance use”) may exacerbate their long-term morbidity and mortality.^4^^,^^7^ Although substance use is often considered developmentally normative in young adulthood,^4^^,^^8^ its consequences are particularly concerning in this medically vulnerable population, where even occasional use has been associated with risk of malignancies and other late effects.^8^, ^9^, ^10^

Neurocognitive impairment, a well-documented late effect of pediatric cancer treatment, may contribute to substance use behaviours. Up to 60% of survivors experience deficits in attention, memory, processing speed, and executive functioning.^11^, ^12^, ^13^, ^14^, ^15^, ^16^, ^17^ This may lead to challenges with inhibitory control, evaluation of risks, and future-oriented decision-making, increasing susceptibility to immediate reward-seeking behaviours, such as smoking or drinking.^8^^,^^12^^,^^13^^,^^15^^,^^18^ Previous studies have demonstrated deficits within these neurocognitive domains were associated with substance use in both adolescent and adult survivors.^8^^,^^10^

Emotional distress and pain are also prevalent among survivors^19^, ^20^, ^21^ and may further compound risk for substance use among survivors with neurocognitive impairment.^9^^,^^22^ The interplay between neurocognitive impairment and psychological distress or pain remains underexplored in survivors; however, there are several plausible biobehavioural mechanisms that may explain susceptibility. Behaviourally, survivors with neurocognitive deficits experience challenges with problem-solving, emotion regulation, or other adaptive coping responses,^23^ increasing their likelihood of substance use to regulate negative affectivity. Neurobiologically, emotional distress and the ongoing experience of pain can further tax cognitive resources, including inhibitory control supported by the prefrontal cortex,^24^^,^^25^ compounding existing neurocognitive vulnerabilities. Clarifying how specific domains of neurocognitive impairment interact with emotional distress and pain in relation to substance use risk may reveal critical targets for intervention and inform strategies to improve long-term survivorship outcomes.

Accordingly, this study aimed to examine associations between neurocognitive impairment (primary exposure) and substance use (primary outcome) among survivors of childhood cancer and to investigate whether emotional distress and pain moderated associations between neurocognitive impairment and substance use. It was hypothesised that neurocognitive impairment would be associated with substance use, and that distress and pain would moderate associations between neurocognitive impairment and substance use. Specifically, in the context of neurocognitive impairment, greater distress and pain symptoms would exacerbate substance use. If supported, these findings may inform targeted screening and intervention approaches to reduce harmful substance use and improve health trajectories in this vulnerable population.

## Methods

### Study design and population

The Childhood Cancer Survivor Study (CCSS) is a retrospectively constructed, multi-institutional cohort study of 5-year survivors of childhood cancer with longitudinal prospective follow-up. Detailed methodology and study design have been described previously.^26^^,^^27^ Survivors in this study were diagnosed with cancer between 1970 and 1999 at one of the CCSS institutions, younger than 21 years at diagnosis, at least 18 years old at survey assessment, and survived at least 5 years after diagnosis. CCSS participants who responded to survey items querying alcohol and tobacco use, and questionnaires regarding neurocognitive and emotional symptoms were included in this study.

### Ethics

CCSS primary site IRB was approved by each of the 31 institutions (IRB Protocol # CR00007578, University of Minnesota), and informed consent was obtained before participants engagement in the study. The CCSS cohort study is registered with ClinicalTrials.gov, NCT01120353.

### Procedures

Data for the present cross-sectional analyses were drawn from CCSS follow-up questionnaires administered between 2003 and 2007. During these follow-up assessments, survivors completed self-report questionnaires evaluating neurocognitive functioning, emotional distress, pain, and health behaviours, including tobacco and alcohol use. Surveys were administered primarily via mailed paper questionnaires, with telephone and web-based completion options available in accordance with CCSS data collection procedures. Medical information and treatment history were extracted from medical records.

Diagnosis and treatment details were abstracted from medical records at each CCSS institution. For survivors treated with radiation therapy, the maximum delivered target dose for each of four intracranial brain segments was determined by summing the contributions from all overlapping fields within that segment.^28^ Cranial radiation dose was then defined as the highest dose among the four segments.

Neurocognitive impairment was assessed with the 25-item CCSS-Neurocognitive Questionnaire (NCQ), that provides scores for four neurocognitive domains: Task Efficiency, Emotional Regulation, Organization, and Memory.^29^ Scores were converted to T-scores based on population norms, with T-scores ≥ 63 considered corresponding to ≥90th percentile of impairment observed in the sibling sample. This threshold was selected to identify clinically meaningful elevations in neurocognitive symptoms relative to sibling norms, rather than normative variability.

Emotional distress was assessed using the 18-item Brief Symptom Inventory (BSI-18), a validated questionnaire measuring acute symptoms of Depression, Anxiety, and Somatization.^30^^,^^31^ Scores were converted to T-scores based on population norms, with T-scores ≥ 63 considered clinically elevated.^32^ Participants who reported taking anxiolytics or antidepressants were also categorised as having anxiety or depression, respectively.

Pain was assessed by self-report in questionnaires, rated by participants as mild, moderate, severe, very severe, or no pain. Pain was dichotomised as present (mild, moderate, severe, very severe) or no pain to reflect clinically relevant symptom burden and to ensure interpretability and adequate power in interaction models.

Tobacco and alcohol use were assessed from responses to questions regarding frequency and quantity of use and categorised as never/past and or current smoking (exposure to at least 100 cigarettes and reports currently smoking).^33^

Alcohol use was obtained from self-report and categorised as never, occasional (≥1 drinks in the past year), risky (>3 drinks per day or 7 drinks per week for women, and >4 drinks per day or 14 drinks per week for men), or heavy drinking (≥5 drinks per day for women and ≥6 drinks per day for men at least once per month for the past year based on current thresholds).^34^

### Statistics

Frequency distributions were used to describe the sample. Covariates were selected a priori based on existing literature and conceptual relevance to both neurocognitive impairment and substance use.^4^^,^^22^^,^^35^, ^36^, ^37^ Because survivors were diagnosed and enrolled over a wide calendar period and completed questionnaires at varying ages, age was modelled using restricted cubic splines with five knots (20, 25, 30, 35, and 45 years) to allow flexible adjustment without imposing linearity. This approach reduces residual confounding by age while preserving interpretability of the main associations. In addition, as a result of under-sampling of acute lymphoblastic leukaemia survivors, all analyses incorporated CCSS sampling weights to account for differential probabilities. Robust (sandwich) standard errors were used for variance estimation.

Differences in substance use between survivors with and without neurocognitive impairment, were assessed using (multinomial) polytomous logistic regression adjusting for identified sociodemographic (sex, race/ethnicity, education, income, marital status) and treatment history (surgery, antimetabolites, anthracyclines, alkylating agents, corticosteroids, cranial radiation), stimulants use, age at cancer diagnosis, and relapse/SMN status.

Polytomous logistic regression models were used to determine if associations between neurocognitive impairment and substance use were moderated by distress or pain. Each interaction term between distress (i.e., depression, anxiety, somatisation)/pain and neurocognitive impairment domains were evaluated separately in each substance-use model. No alcohol use served as the reference category for models examining alcohol use. For tobacco use models, never/past smoking served as the reference category. Odds ratios represent the relative odds of membership in each substance use category compared with the reference group.

All analyses were performed using SAS software (version 9.4; SAS Institute Inc., Cary, NC, USA).

### Role of funding source

No funding body was involved in the development of the study design, in data collection, analyses, or interpretation of the data, in the drafting of the manuscript, or in the decision to submit the paper for publication.

## Results

This study included 11,151 survivors who were on average 31.4 (SD 7.5) years of age at time of assessment, largely non-Hispanic white (87.3%; n = 9445/11,151) and college educated (85.8% some college or college graduate; n = 9555/11,151). Survivors reported former (14.6%; n = 1482/10,182) or current smoking (13.7%; n = 1395/10,182) and reported either occasional (39.3%; n = 3901/9894), risky (40.9%; n = 4059/9894), or heavy (11%; n = 1096/9894) alcohol use. Clinically elevated anxiety, depression, and somatisation symptoms were observed in 7.6% (n = 838/11,118), 11.7% (n = 1276/11,123), and 11.3% (n = 1228/11,120) of survivors, respectively. Presence of pain symptoms were reported by 23% (n = 2556/11,089) of survivors. These and other sociodemographic, diagnosis, and treatment data are described in Table 1 and Supplementary Table S1.

**Table 1:** Demographic and treatment characteristics of survivors of childhood cancer overall and by substance use.^a^

	All survivors	Cigarette use	Alcohol use		
	(N = 11,151)	Current Smoking (N = 1395)	Occasional (N = 3901)	Risky (N = 4059)	Heavy (N = 1096)
	N (%)	N (%)	N (%)	N (%)	N (%)
Age at diagnosis					
<1	689 (5.5)	95 (6.3)	179 (4.2)	274 (5.9)	95 (7.4)
1–4	3202 (31.9)	417 (31.1)	967 (27.2)	1168 (32.5)	342 (35.5)
5–9	2530 (23.9)	318 (24.8)	852 (23.6)	936 (24.4)	255 (25.3)
10–14	2589 (21.4)	328 (22.1)	967 (23.1)	966 (21.7)	226 (17.9)
15–21	2141 (17.3)	237 (15.7)	936 (21.9)	715 (15.5)	178 (14.0)
Age at questionnaire (Median (IQR))	30.9 (10.3)	31.0 (10.1)	32.5 (10.4)	30.3 (9.5)	29.0 (9.7)
Sex					
Female	5918 (53.2)	709 (51.7)	2295 (59.0)	2120 (52.6)	437 (40.1)
Male	5233 (46.8)	686 (48.3)	1606 (41.0)	1939 (47.4)	659 (59.9)
Race/Ethnicity					
White non-Hispanic	9445 (87.3)	1214 (89.8)	3310 (87.2)	3455 (87.6)	941 (87.9)
Black non-Hispanic	385 (3.8)	27 (2.0)	145 (4.1)	122 (3.3)	28 (2.7)
Hispanic	619 (6.0)	65 (5.6)	204 (5.8)	241 (6.5)	64 (6.4)
Other	283 (2.9)	33 (2.6)	106 (2.9)	89 (2.7)	24 (3.0)
Missing	419	56	136	152	39
Education					
<12 yrs	326 (3.0)	118 (8.5)	109 (3.0)	79 (2.0)	32 (2.9)
High school graduate	1270 (11.2)	274 (19.4)	372 (9.3)	374 (9.1)	115 (11.2)
Some college	3728 (33.4)	634 (46.1)	1203 (30.8)	1293 (31.7)	435 (39.7)
College graduate	5827 (52.4)	369 (26.0)	2217 (56.9)	2313 (57.2)	514 (46.2)
Annual household income					
<$19,999	1318 (13.3)	304 (24.5)	440 (12.4)	423 (11.7)	130 (12.5)
$20,000–39,999	2162 (20.9)	398 (29.8)	732 (19.9)	749 (19.4)	223 (22.0)
$40,000–59,999	1940 (18.8)	255 (19.8)	752 (20.7)	666 (17.3)	177 (17.1)
Over $60,000	4871 (47.0)	342 (25.9)	1735 (47.0)	2000 (51.5)	505 (48.4)
Marital/Relationship status					
Never married	4872 (45.2)	606 (43.8)	1454 (38.4)	1762 (45.3)	544 (51.8)
Married/living with partner	5474 (48.2)	560 (40.3)	2177 (55.3)	2020 (48.3)	458 (40.5)
Widowed/divorced/separated	761 (6.6)	222 (15.9)	260 (6.3)	264 (6.4)	87 (7.7)
Treatment Characteristics					
Surgery					
Yes	8327 (72.1)	1024 (73.7)	2936 (73.1)	3055 (70.5)	807 (68.8)
No	2307 (27.9)	286 (26.3)	813 (26.9)	855 (29.5)	242 (31.2)
Chemotherapy					
Antimetabolites					
Yes	4616 (49.7)	576 (48.8)	1645 (49.1)	1705 (51.3)	464 (52.9)
No	6025 (50.3)	734 (51.2)	2108 (50.9)	2206 (48.7)	587 (47.1)
Anthracyclines					
Yes	5032 (51.5)	579 (48.5)	1772 (51.1)	1942 (54.4)	519 (53.4)
No	5585 (48.5)	729 (51.5)	1972 (48.9)	1964 (45.6)	528 (46.6)
Alkylating agents					
Yes	5551 (52.7)	625 (48.5)	2017 (54.2)	2020 (52.2)	536 (51.8)
No	5049 (47.3)	680 (51.5)	1724 (45.8)	1881 (47.8)	509 (48.2)
Corticosteroids					
Yes	4526 (48.3)	538 (45.9)	1605 (47.5)	1709 (50.5)	452 (51.2)
No	6115 (51.7)	772 (54.1)	2148 (52.5)	2202 (49.5)	599 (48.8)
Radiation					
Yes	6081 (53.3)	707 (51.3)	2268 (57.0)	2037 (48.4)	520 (43.8)
No	4581 (46.7)	608 (48.7)	1493 (43.0)	1877 (51.6)	533 (56.2)
Cranial radiation					
None/Scatter (≤2 Gy)	7505 (72.5)	963 (74.4)	2592 (71.4)	2893 (75.2)	815 (80.1)
>2 Gy to <20 Gy	1036 (10.8)	129 (10.8)	344 (10.3)	395 (11.4)	88 (8.7)
≥20 Gy	1894 (16.7)	203 (14.8)	723 (18.4)	562 (13.5)	129 (11.2)
Cancer diagnosis					
Leukaemia	3365 (37.9)	440 (37.1)	1153 (36.3)	1226 (39.6)	339 (41.4)
CNS malignancy	1704 (13.6)	174 (11.5)	601 (13.9)	592 (12.6)	128 (9.9)
Hodgkin lymphoma	1529 (12.2)	178 (11.7)	603 (14.0)	554 (11.8)	138 (10.7)
Non-Hodgkin lymphoma	941 (7.5)	128 (8.4)	353 (8.2)	347 (7.4)	88 (6.8)
Neuroblastoma	730 (5.8)	105 (6.9)	215 (5.0)	277 (5.9)	96 (7.4)
Wilms	1037 (8.3)	143 (9.4)	298 (6.9)	427 (9.1)	110 (8.5)
Soft tissue sarcoma	835 (6.7)	96 (6.3)	307 (7.1)	286 (6.1)	78 (6.0)
Osteosarcoma	600 (4.8)	80 (5.3)	225 (5.2)	206 (4.4)	69 (5.3)
Other bone tumours	410 (3.3)	51 (3.4)	146 (3.4)	144 (3.1)	50 (3.9)

a All statistics except counts (N) are weighted for under-sampling of acute lymphoblastic leukaemia (ALL) in the the CCSS cohort. Reference category for alcohol use models: never drinkers. Reference category for cigarette use models: never/past smoking.

The proportion of survivors with neurocognitive impairment and emotional distress or pain reporting substance use behaviours are shown in Supplementary Tables S2 and S3 In multivariable models adjusted for sociodemographic factors and treatment factors, survivors with impaired task efficiency were significantly less likely to be current smokers (odds ratio [OR] 0.80, 95% CI 0.66–0.97; p = 0.022) and engage in occasional (0.70, 0.56–0.87; p = 0.0020), risky (0.60, 0.48–0.75; p < 0.001), or heavy (0.63, 0.47–0.84; p = 0.0020) drinking (Table 2). Conversely, survivors with impaired emotion regulation were significantly more likely to be current smokers (1.81, 1.53–2.14; p < 0.0001) and engage in occasional (1.40, 1.10–1.79; p = 0.0070), risky (1.59, 1.25–2.03; p < 0.0001), or heavy (2.44, 1.84–3.23; p < 0.0001) drinking. Similarly, survivors who reported memory problems were more likely to engage in occasional (1.38, 1.10–1.73; p = 0.0060) or risky drinking (1.37, 1.09–1.71; p = 0.0070; Table 2).

**Table 2:** Multivariable polytomous model examining neurocognitive impairment and substance use behaviours in survivors of childhood cancer.

Variables	Cigarette use (N = 8340)		Alcohol use (N = 8278)					
	Current smoking		Occasional		Risky		Heavy	
	OR (95% CI)	p	OR (95% CI)	p	OR (95% CI)	p	OR (95% CI)	p
Neurocognitive concerns								
Task efficiency								
Impaired	0.80 (0.66–0.97)	0.022	0.70 (0.56–0.87)	0.0020	0.60 (0.48–0.75)	<0.001	0.63 (0.47–0.84)	0.0020
Not impaired (reference)								
Emotion Regulation								
Impaired	1.81 (1.53–2.14)	<0.001	1.40 (1.10–1.79)	0.0070	1.59 (1.25–2.03)	<0.001	2.44 (1.84–3.23)	<0.0001
Not impaired (reference)								
Organization								
Impaired	1.21 (0.99–1.49)	0.059	0.96 (0.73–1.26)	0.76	0.90 (0.69–1.18)	0.46	1.19 (0.86–1.64)	0.29
Not impaired (reference)								
Memory								
Impaired	1.14 (0.96–1.36)	0.13	1.38 (1.10–1.73)	0.0060	1.37 (1.09–1.71)	0.0070	1.04 (0.78–1.38)	0.79
Not impaired (reference)								

Analyses accounted for under-sampling of acute lymphoblastic leukaemia (ALL) in the CCSS cohort using weighted polytomous logistic regression; analyses were also adjusted for cubic splines (five knots at 20, 25, 30, 35 and 45 years) of age at questionnaire. Models were adjusted for sociodemographic factors, treatment exposures, relapse, and secondary malignancy status. Models were performed separately for cigarette use and alcohol use. Reference category for alcohol use models: no use. Reference category for cigarette use models: never/past use.

**Fig. 1:**
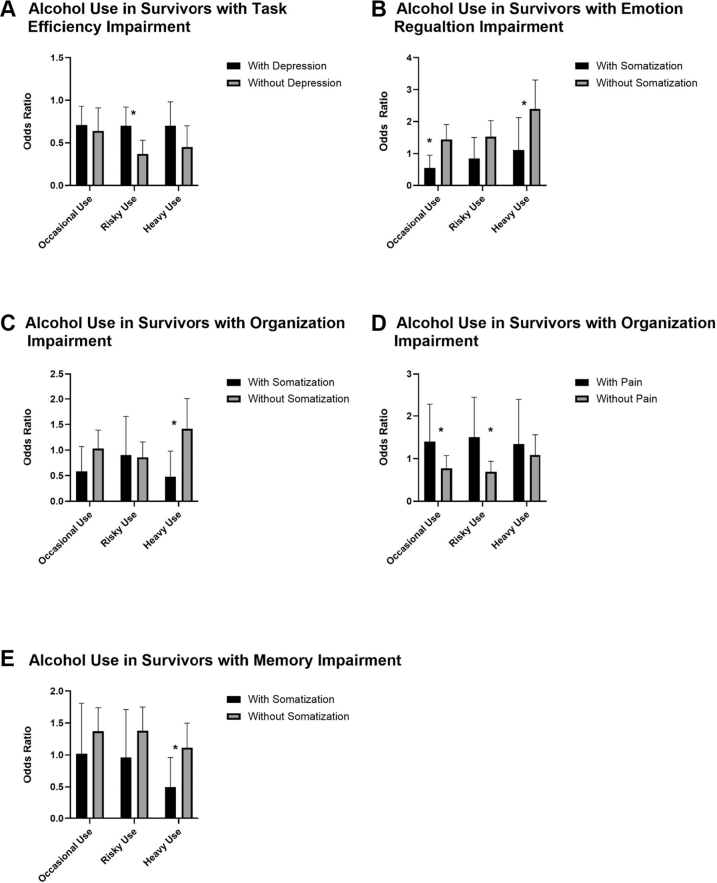
Associations between substance use and domains of neurocognitive impairment moderated by psychological distress and pain. For models A, B, C, D, and E, analyses accounted for under-sampling of acute lymphoblastic leukaemia in the CCSS cohort using weighted polytomous logistic regression; analyses were also adjusted for cubic splines (five knots at 20, 25, 30, 35 and 45 years) of age at questionnaire. Reference category for alcohol use models: no use.

Fig. 1 and Supplementary Tables S4–S8 highlight findings from moderation analyses examining psychological distress and pain with neurocognitive impairment on substance use categories. While survivors with impaired task efficiency had reduced likelihood of substance use, elevated depressive symptoms appeared to attenuate the association between task efficiency impairment on odds of risky alcohol use (with depression vs. without OR 0.70 [95% CI, 0.54–0.92] vs. 0.37 [0.26–0.53]; p = 0.0030; Fig. 1A), although risk remained reduced compared to survivors without impaired task efficiency. Elevated symptoms of somatisation reversed the direction of the association of emotional regulation impairment with odds of occasional alcohol use (with somatisation vs. without OR 0.54 [0.30–0.95] vs. 1.44 [1.08–1.91]; p = 0.0021; Fig. 1B) and attenuated the association of emotional regulation impairment on odds of heavy alcohol use (with somatisation vs. without 1.10 h[0.57–2.12] vs. 2.39 [1.73–3.30]; p = 0.030; Fig. 1C). Elevated symptoms of somatisation again reversed the association of impaired organisation with odds on risk for heavy alcohol use (with somatisation vs. without OR 0.48 [0.23–0.98] vs. 1.42 [1.00–2.01]; p = 0.0060). Pain moderated the association of impaired organisation with odds of risky alcohol use (with pain vs. without OR 1.50 [0.92–2.44] vs. 0.69 [0.50–0.94]; p = 0.0070; Fig. 1D). Elevated symptoms of somatisation also negated the association of reduced odds of heavy drinking with memory impairment (with memory impairment vs. without OR 0.50 [0.26–0.96] vs. 1.11 [0.83–1.50]; p = 0.021; Fig. 1E).

## Discussion

This study offers several novel insights into the complex interplay between neurocognitive impairment, psychological distress, pain, and substance use among adult survivors of childhood cancer. Notably, the present findings demonstrate that the presence or absence of neurocognitive impairment alone is not always sufficient to understand risk for substance use and that the type of impairment in the context of emotional and physical functioning may modify substance use risk. For example, survivors with task efficiency impairments were less likely to engage in substance use; however, the presence of depression appeared to attenuate this finding. In contrast, survivors with memory and emotion regulation impairments were at elevated risk for substance use, but the presence of somatisation reduced substance use risk. These findings highlight the importance of considering the broader psychosocial context in which neurocognitive impairments occur for understanding risky health behaviour engagement in this vulnerable population.

In contrast to our hypothesis that impaired task efficiency would be associated with greater risk for substance use, impaired task efficiency was associated with reduced likelihood of smoking and some alcohol use behaviours. While challenges with task efficiency are often assumed to increase risk for poor decision-making,^36^ our findings suggest a more nuanced relationship. Survivors with task efficiency impairments, particularly those exposed to high does cranial radiation, differ systematically in functional and social status, which could influence substance use behaviours and should be explored in future research. However, among survivors with task efficiency impairments, depression attenuated the negative association between heavy alcohol use and task efficiency impairment. This underscores the importance of screening for and treating depression in survivorship care, as depressive symptoms may indicate heightened risk for substance use among survivors with cognitive limitations. Interestingly, depression and anxiety did not significantly moderate associations between other neurocognitive domains and substance use, potentially suggesting that the impact of emotional distress may be domain specific.

Survivors with impairments in emotion regulation, which reflects challenges managing emotional experiences vs. emotional distress, which is one’s own subjective experience of negative emotions (e.g., depression, anxiety) and memory were at higher risk for cigarette and alcohol use. However, somatisation (e.g., fatigue, gastrointestinal issues related to psychological distress) appeared to reverse associations between these neurocognitive impairments and certain substance use behaviours. This contrasts with research focused on non-cancer populations, which demonstrates a greater incidence of substance use in the context of somatic distress.^38^ Among survivors of childhood cancer, the high prevalence of chronic health conditions^1^ and functional impairments,^39^ commonly associated with somatic symptoms, are often linked to increased healthcare use and touch points with healthcare providers,^40^ which may offer a protective benefit against substance use. This divergence underscores the distinct survivorship context in which long-term follow-up may not only mitigate the physical consequences of somatic symptoms but also buffer against maladaptive coping behaviours such as substance use.

Pain, a known risk factor for substance use,^22^^,^^38^ also moderated associations between neurocognitive impairment and alcohol use. Specifically, survivors with impaired organisation and pain were more likely to report occasional and risky drinking. Impaired organisation, which is characterised by challenges with planning, sequencing, and goal directed behaviour may reduce a survivor’s ability to manage daily stressors (e.g., pain) and leverage effective pain management coping strategies, leading survivors to engage in more immediate, short-term relief. Given the high prevalence of pain symptoms among cancer survivors^21^ and their intersection with cognitive and behavioural vulnerabilities, integrating behavioural pain management strategies into routine survivorship care is essential.

These findings demonstrate the importance of moving beyond universal survivorship screening toward risk-stratified approaches that integrate domain specific neurocognitive vulnerabilities with co-occurring distress and pain symptoms. Survivors with impairment in emotional regulation, memory, or organisation, particularly when accompanied by pain or somatic distress, may warrant enhanced monitoring for substance use and targeted preventive intervention, rather than reliance on general health behaviour counselling alone. Given evidence that neurocognitive and psychosocial assessments and related supportive services remain underused in survivorship and late-effects clinics,^41^ these data highlight an opportunity to better leverage existing guidelines by using cognitive-psychosocial risk profiles to guide intervention prioritisation and referral.

Survivors with emotional regulation difficulties and pain symptomology may benefit from interventions that enhance distress tolerance as well as pain- and adaptive-related coping strategies, such as cognitive-behavioural therapy or mindfulness-based approaches.^42^, ^43^, ^44^ For those with cognitive impairments and co-occurring pain or depression, coordinated care that includes neurocognitive rehabilitation and symptom management may mitigate risk.^45^ Embedding these targeted interventions within survivorship programs may reduce long-term health risks and improve quality of life for this vulnerable population.

A major strength of this study is the large, well-characterised CCSS cohort, which enables examination of biobehavioural pathways across decades of survivorship and includes meaningful absolute representation of racially and ethnically minoritised survivors from the USA and Canada. Although previous research demonstrates that childhood cancer survivors exhibit lower cigarette use to the general population and similar or greater alcohol use compared to the general population across Western and Western-European cohorts.^4^, ^5^, ^6^, ^7^^,^^46^^,^^47^ rates of substance use in Asian cohorts appear to be much lower.^48^ Thus, while the present study extends this literature in Western-European populations by moving beyond prevalence comparisons to identify within-survivor risk profiles and insights to inform targeted screening, these findings may not be applicable to all childhood cancer survivorship populations. Further, the cohort’s composition, which includes predominantly non-Hispanic White survivors treated in earlier therapeutic eras with different toxicity profiles, supportive care practices, and survivorship guidelines, may limit generalisability to contemporary, more diverse survivor populations. For example, current definitions of alcohol consumption were applied to a retrospective cohort, and changes in public health guidelines and social norms surrounding alcohol use over time may have differentially influenced drinking behaviours across eras, potentially introducing exposure misclassification. Additionally, findings may not fully capture substance use patterns and behaviours (e.g., vaping) relevant to contemporary survivorship populations. While distress, neurocognitive difficulties, and pain remain common among more recently treated survivors,^49^^,^^50^ suggesting that the mechanisms identified here remain clinically relevant to contemporary cohorts, future research should expand this research to more diverse and contemporarily treated survivors.

Self-reporting of primary exposure and outcome variables also presents as a limitation to the current study’s findings. The CCSS-Neurocognitive Questionnaire is a validated tool^29^; however, reliance on self-report introduces potential bias, particularly in a population where cognitive impairments, such as memory deficits, may affect the accuracy of responses. Further, social desirability or stigma may have led to underreporting of substance use or mental health symptoms, potentially attenuating observed associations. Nevertheless, these measures reflect patient-reported outcomes that are routinely used and feasible in survivorship clinic settings, capturing symptom burden and functional concerns that are directly relevant to clinical screening and care.

Although models adjusted for relevant demographic and treatment-related factors, residual confounding remains possible due to unmeasured characteristics that may be independently associated with both neurocognitive impairment and substance use including neighbourhood socioeconomic factors, comorbid psychiatric conditions, and other unassessed functional limitations. Further, the cross-sectional design precludes causal inference, and findings should therefore be interpreted with these limitations in mind. Finally, pain was dichotomised as present vs. absent to reflect clinically relevant symptom burden; however, this approach does not capture heterogeneity in pain severity, chronicity, or management, which may differentially influence substance use behaviours. Nevertheless, the large, well-characterised cohort represents a key strength, supporting a nuanced examination of associations between neurocognitive impairment, co-occurring distress and pain, and substance use among long-term survivors. Future research should leverage longitudinal models, incorporating both subjective and objective assessments in more diverse populations, and should consider a broader range of substance use behaviours (e.g., vaping, prescription misuse, marijuana)^22^^,^^35^^,^^51^ relevant to pediatric cancer survivors.

Taken together, this study highlights the nuanced and domain-specific associations between neurocognitive impairment, emotional distress, and substance use among adult survivors of childhood cancer. Emotional regulation and memory impairments emerged as risk factors for cigarette and alcohol use, particularly in the presence of pain symptomology, while task efficiency impairment was unexpectedly associated with lower substance use. These findings underscore the importance of examining neurocognitive impairments within the broader context of emotional and physical health, as findings suggest that substance use risk among survivors is associated with interactions between cognitive vulnerabilities and co-occurring symptomatology. By identifying survivors at highest risk and addressing modifiable cognitive and emotional vulnerabilities, clinicians can more effectively mitigate long-term health risks and promote healthier trajectories across the lifespan.

## Contributors

Conceptualisation: EC, TS, MMY, MC, KRK. Data Curation, Formal Analysis, and Data Verification: WQ, EC, KE, YY, RD. WQ, EC, KE, YY, WL, and RD had full access to the study data throughout the analysis phase. Methodology: WQ, YY, EC, KE. Supervision: KE. Writing - original draft: RW, EC, WQ, KE. Writing - review & editing: RW, EC, WQ, KE, RD, TS, MMY, MC, KK, PN, WL, GTA, RH, TMB, WL. Resource acquisition: GTA. All authors read and approved the final version of the manuscript.

## Data sharing statement

The Childhood Cancer Survivor Study is a US National Cancer Institute funded resource (U24 CA55727) to promote and facilitate research among long-term survivors of cancer diagnosed during childhood and adolescence. CCSS data are publicly available on dbGaP at https://www.ncbi.nlm.nih.gov/gap/ through its accession number phs001327.v2.p1, and on the St Jude Survivorship Portal within the St. Jude Cloud at https://survivorship.stjude.cloud/. Use of the CCSS data that leverages the expertise of CCSS Statistical and Survivorship research and resources will be considered on a case-by case basis. For this use, a research Application of Intent followed by an Analysis Concept Proposal must be submitted for evaluation by the CCSS Publications Committee. Users interested in using this resource are encouraged to visit http://ccss.stjude.org. Full analytical data sets associated with CCSS publications since January of 2023 are also available on the St. Jude Survivorship Portal at https://viz.stjude.cloud/community/cancer-survivorship-community∼4/publications.

## Declaration of interests

RMH reports funding from the National Institutes of Health/National Cancer Institute (NIH/NCI) Childhood Cancer Survivor Study (CCSS; U24CA055727; PI: Gregory T. Armstrong; 12/1/2021–11/30/2026) to conduct radiation dosimetry for CCSS.

YY reports funding as an investigator or collaborator from the National Cancer Institute (U24CA055727, PI: Armstrong; P30CA021765, PI: Roberts; U01CA195547, MPI: Hudson/Ness; R01CA270157, MPI: Bhakta/Yasui; R01CA258193, MPI: Huang/Yasui; R01CA261750-01A1, MPI: Howell/Mulrooney/Yasui; R01CA261898, MPI: Burridge/Sapkota), NIH & MD Anderson Cancer Center, Northwestern University, the American Lebanese Syrian Associated Charities, and support from NCI training grant T32CA225590 (PI: Krull).

KK reports funding from the National Cancer Institute.

All other authors declare no competing interests.
