# Supplementary material for: Neurocognitive impairment and substance use in adult survivors of childhood cancer: a cross-sectional analysis from the Childhood Cancer Survivor Study
Source: eClinicalMedicine. 2026 Apr 29;95:103924. doi: 10.1016/j.eclinm.2026.103924 (PMC13141803; doi:10.1016/j.eclinm.2026.103924)
Supplement: Supplementary Tables S1–S8 [file mmc1.docx]

|  | Survivors (N=11,151) |
| --- | --- |
|  | *N (%)* |
| Neurocognitive Concerns |  |
| Task Efficiency |  |
| Impaired | 2485 (22.2) |
| T-score (Mean (SD)) | 53.5 (13.6) |
| Emotion Regulation |  |
| Impaired | 2084 (18.2) |
| T-score (Mean(SD)) | 51.8 (11.8) |
| Organization |  |
| Impaired | 1306 (11.5) |
| T-score (Mean(SD)) | 50.5 (11.1) |
| T-score (Median (IQR)) | 47.7 (13.1) |
| Memory |  |
| Impaired | 2724 (24.5) |
| T-score (Mean(SD)) | 52.9 (12.8) |
| Cigarette Use |  |
| Never smoker | 7305 (71.4) |
| Past smoker | 1482 (14.6) |
| Current smoker | 1395 (13.7) |
| Missing | 969 |
| Alcohol Use |  |
| No use | 865 (8.7) |
| Occasional use | 3901 (39.3) |
| Risky use | 4059 (40.9) |
| Heavy use | 1096 (11.0) |
| Missing | 1230 |
| Distress |  |
| Anxiety |  |
| Yes | 838 (7.6) |
| No | 10280 (92.4) |
| Missing | 33 |
| T-score (Mean(SD)) | 46.9 (9.9) |
| Depression |  |
| Yes | 1276 (11.7) |
| No | 9847 (88.3) |
| Missing | 28 |
| T-score (Mean(SD)) | 48.6 (10.2) |
| Somatization |  |
| Yes | 1288 (11.3) |
| No | 9832 (88.7) |
| Missing | 31 |
| T-score (Mean(SD)) | 49.3 (9.3) |
| Pain |  |
| No pain | 8533 (77.0) |
| Mild | 1404 (12.7) |
| Moderate | 743 (6.7) |
| Severe | 308 (2.8) |
| Very Severe | 101 (0.9) |
| Missing | 62 |

Supplemental Table 1| Descriptive statistics among primary outcome and predictor variables*

*All statistics except counts (N) were accounted were weighted for under-sampling of acute lymphoblastic leukemia (ALL) in the CCSS cohort.

## Supplemental Table 2| Unadjusted models examining mean, standard deviation, and impairment across alcohol use domains in survivors of childhood cancer.

|  | Mean (SD) | No Use  (N=865) | Occasional Use  (N=3901) | Risky Use  (N=4059) | Heavy Use  (N=1096) | p-value |
| --- | --- | --- | --- | --- | --- | --- |
| Mean (SD) for NCQ Scores Across Alcohol Use Domains | 1. Task Efficiency | 56.1 (15.0) | 53.5 (13.5) | 52.6 (13.1) | 53.7 (13.5) | <0.001 |
|  | 2. Emotion Regulation | 50.4 (12.0) | 51.4 (11.5) | 51.6 (11.9) | 53.3 (12.5) | <0.001 |
|  | 3. Organization | 50.4 (11.6) | 50.5 (10.9) | 50.1 (11.0) | 50.9 (11.7) | 0.94 |
|  | 4. Memory | 52.8 (13.1) | 53.2 (12.9) | 52.4 (12.9) | 53.0 (12.4) | 0.27 |
| Number and % of Survivors with NCQ Impairment Across Alcohol Use Domains | 1. Task Efficiency | 266 (29.6) | 866 (22.0) | 788 (19.7) | 249 (22.8) | <0.001 |
|  | 2. Emotion Regulation | 152 (16.4) | 682 (16.8) | 710 (17.2) | 243 (22.3) | <0.001 |
|  | 3. Organization | 105 (12.0) | 450 (11.5) | 433 (10.4) | 152 (13.6) | 0.010 |
|  | 4. Memory | 232 (25.7) | 995 (25.5) | 914 (23.2) | 253 (23.2) | 0.041 |
| Mean (SD) for BSI Scores Across Alcohol Use Domains | 1. Anxiety | 45.3 (9.6) | 46.7 (9.6) | 46.8 (10.0) | 48.3 (10.7) | <0.001 |
|  | 2. Somatization | 48.5 (9.3) | 49.5 (9.3) | 48.6 (9.0) | 49.9 (9.7) | 0.55 |
|  | 3. Depression | 47.5 (10.1) | 48.0 (9.8) | 48.5 (10.3) | 50.4 (11.3) | <0.001 |
| Number and % of Survivors with Clinically Elevated BSI Scores Across Alcohol Use Domains | 1. Anxiety | 45 (5.3) | 258 (6.8) | 300 (7.5) | 107 (9.6) | <0.001 |
|  | 2. Somatization | 88 (9.5) | 457 (11.7) | 377 (9.0) | 145 (13.4) | <0.001 |
|  | 3. Depression | 85 (9.8) | 398 (10.3) | 456 (11.5) | 158 (15.4) | <0.001 |
| Mean (SD) for Pain Scores Across Alcohol Use Domains | 1. Pain Present | 1.3 (0.8) | 1.4 (0.9) | 1.3 (0.8) | 1.3 (0.8) | 0.010 |
| Number and % of Survivors with Pain Present Across Alcohol Use Domains | 1. Pain Present | 194 (22.0) | 946 (24.2) | 852 (20.5) | 244 (20.9) | <0.001 |

^*^ All statistics except counts (N) were weighted for under-sampling of acute lymphoblastic leukemia (ALL) in the CCSS cohort

^ⱡ^ For cells with count less than 5, p values were obtained from Fisher’s Exact test

## Supplemental Table 3| Unadjusted models examining mean, standard deviation, and impairment never, past, and cigarette use in survivors of childhood cancer.

|  | Mean (SD) | Never  (N=7305) | Past  (N=1482) | Current  (N=1395) | p-value |
| --- | --- | --- | --- | --- | --- |
| Mean (SD) for NCQ Scores Across Cigarette Use Domains | 1. Task Efficiency | 53.1 (13.2) | 53.4 (13.4) | 56.5 (14.7) | <0.0001 |
|  | 2. Emotion Regulation | 50.9 (11.4) | 53.6 (11.8) | 56.3 (12.2) | <0.0001 |
|  | 3. Organization | 50.4 (10.8) | 50.8 (11.1) | 52.4 (11.7) | <0.0001 |
|  | 4. Memory | 52.2 (12.3) | 53.5 (12.8) | 55.9 (13.9) | <0.0001 |
| Number and % of Survivors with NCQ Impairment Across Cigarette Use Domains | 1. Task Efficiency | 1568 (21.4) | 324 (21.6) | 393 (28.9) | <0.0001 |
|  | 2. Emotion Regulation | 1188 (15.6) | 334 (22.9) | 423 (31.2) | <0.0001 |
|  | 3. Organization | 805 (10.9) | 172 (11.7) | 235 (16.7) | <0.0001 |
|  | 4. Memory | 1650 (22.5) | 393 (27.0) | 449 (33.2) | <0.0001 |
| Mean (SD) for BSI Scores Across Cigarette Use Domains | 1. Anxiety | 46.1 (9.2) | 48.1 (10.3) | 50.8 (11.2) | <0.0001 |
|  | 2. Somatization | 48.7 (8.7) | 50.4 (9.8) | 52.6 (10.5) | <0.0001 |
|  | 3. Depression | 47.9 (9.6) | 49.3 (10.4) | 52.6 (11.6) | <0.0001 |
| Number and % of Survivors with Clinically Elevated BSI Scores Across Cigarette Use Domains | 1. Anxiety | 395 (5.5) | 151 (10.1) | 215 (15.7) | <0.0001 |
|  | 2. Somatization | 695 (9.2) | 215 (14.0) | 291 (21.5) | <0.0001 |
|  | 3. Depression | 696 (9.8) | 191 (12.5) | 286 (21.0) | <0.0001 |
| Mean (SD) for Pain Scores Across Cigarette Use Domains | 1. Pain Present | 1.3 (0.8) | 1.5 (1.0) | 1.6 (1.1) | <0.0001 |
| Number and % of Survivors with Pain Present Across Cigarette Use Domains | 1. Pain Present | 1576 (21.1) | 367 (24.8) | 413 (30.2) | <0.0001 |

^*^ All statistics except counts (N) were weighted for under-sampling of acute lymphoblastic leukemia (ALL) in the CCSS cohort

^ⱡ^ For cells with count less than 5, p values were obtained from Fisher’s Exact test

Supplemental Table 4 | Multivariable polytomous logistic regression model examining alcohol use among survivors of childhood cancer – with moderating effects of depression on task efficiency impairment

| **Variables** | **Alcohol Use**  **(N=8245)** | | | | | | | | | | | |  |
| --- | --- | --- | --- | --- | --- | --- | --- | --- | --- | --- | --- | --- | --- |
|  | **Occasional** | | | **Risky** | | | | **Heavy** | | | | |  |
|  | **OR (95% CI)** | ***P*** | | **OR (95% CI)** | | ***P*** | | **OR (95% CI)** | | ***P*** | | |  |
| Neurocognitive concerns |  | | | | | | | | | | | |  |
| Task efficiency |  | | | | | | | | | | | |  |
| Impaired | 0.64 (0.45 - 0.91) | 0.013 | 0.37 (0.26 - 0.53) | | <.0001 | | | | 0.45 (0.28 - 0.70) | | <.0001 | | |
| Not impaired (ref) |  |  |  |  |  |  |  |  |  |  |  |  |  |
| Emotional regulation |  |  |  | |  | | | |  | |  | | |
| Impaired | 1.20 (0.94 - 1.53) | 0.15 | 1.35 (1.06 - 1.72) | | 0.01 | | | | 2.06 (1.54 - 2.74) | | <.0001 | | |
| Not impaired (ref) |  |  |  |  |  |  |  |  |  |  |  |  |  |
| Organization |  | | | | | | | | | | | |  |
| Impaired | 0.92 (0.70 - 1.21) | 0.54 | 0.87 (0.67 - 1.15) | | 0.33 | | | | 1.14 (0.83 - 1.58) | | 0.43 | | |
| Not impaired (ref) |  |  |  |  |  |  |  |  |  |  |  |  |  |
| Memory |  | | | | | | | | | | | |  |
| Impaired | 1.32 (1.05 - 1.65) | 0.017 | 1.31 (1.05 - 1.64) | | 0.02 | | | | 0.97 (0.73 - 1.29) | | 0.85 | | |
| Not impaired (ref) |  |  |  |  |  |  |  |  |  |  |  |  |  |
| Distress |  | | | | | | | | | | | |  |
| Depression |  | | | | | | | | | | | |  |
| Yes | 1.02 (0.83 - 1.26) | 0.87 | 0.95 (0.77 - 1.16) | | 0.60 | | | | 0.82 (0.65 - 1.05) | | | 0.11 | |
| No (ref) |  |  |  |  |  |  |  |  |  |  |  |  |  |
| Anxiety |  | | | | | | | | | | | |  |
| Yes | 1.79 (1.24 - 2.59) | 0.0020 | 1.89 (1.31 - 2.72) | | <.0001 | | | | 1.93 (1.28 - 2.91) | | | 0.002 | |
| No (ref) |  |  |  |  |  |  |  |  |  |  |  |  |  |
| Somatization |  | | | | | | | | | | | |  |
| Yes | 1.28 (0.94 - 1.73) | 0.11 | 1.05 (0.77 - 1.42) | | 0.77 | | | | 1.52 (1.07 - 2.16) | | | 0.011 | |
| No (ref) |  |  |  |  |  |  |  |  |  |  |  |  |  |
| Pain |  | | | | | | | | | | | |  |
| Yes | 1.02 (0.83 - 1.25) | 0.88 | 1.00 (0.81 - 1.22) | | 0.96 | | | | 0.95 (0.75 - 1.22) | | | 0.71 | |
| No (red) |  |  |  |  |  |  |  |  |  |  |  |  |  |
| Task Efficiency*Depression | 1.11 (0.74 - 1.67) | 0.63 | 1.91 (1.25 - 2.90) | | 0.0030 | | | | 1.57 (0.94 - 2.63) | | | 0.085 | |
| **Impaired Task Efficiency**  **with Depression** | 0.71 (0.54 - 0.93) | 0.014 | 0.70 (0.54 - 0.92) | | | | 0.010 | | 0.70 (0.50 - 0.98) | | | 0.038 | |
| **Impaired Task Efficiency without Depression** | 0.64 (0.45 - 0.91) | 0.013 | 0.37 (0.26 - 0.53) | | | | <.0001 | | 0.45 (0.28 - 0.70) | | | <.0001 | |

Analyses were accounted for under-sampling of acute lymphoblastic leukemia (ALL) in the CCSS cohort using weighted polytomous logistic regression; analyses were also adjusted for cubic splines (five knots at 20, 25, 30, 35 and 45 years) of age at questionnaire. Models were adjusted for sociodemographic factors, treatment exposures, relapse, and secondary malignancy status. Ref=reference category Reference category for alcohol use models: no us.e

Supplemental Table 5| Multivariable multinomial logistic regression model examining alcohol use among survivors of childhood cancer – with moderating effects of somatization on emotion regulation

| **Variables** | **Alcohol Use**  **(N=8245)** | | | | | | | | | | | | | |  |
| --- | --- | --- | --- | --- | --- | --- | --- | --- | --- | --- | --- | --- | --- | --- | --- |
|  | **Occasional** | | | **Risky** | | | | **Heavy** | | | | | | |  |
|  | **OR (95% CI)** | ***P*** | | **OR (95% CI)** | | ***P*** | | **OR (95% CI)** | | | ***P*** | | | |  |
| Neurocognitive concerns |  | | | | | | | | | | | | | |  |
| Task efficiency |  | | | | | | | | | | | | | |  |
| Impaired | 0.67 (0.53 - 0.84) | <.0001 | 0.58 (0.46 - 0.73) | | <.0001 | | | | 0.60 (0.44 - 0.80) | | | <.0001 | | |  |
| Not impaired (ref) |  |  |  |  |  |  |  |  |  |  |  |  |  |  |  |
| Emotional regulation |  |  |  | |  | | | |  | | |  | | |  |
| Impaired | 1.44 (1.08 - 1.91) | 0.012 | 1.53 (1.16 - 2.03) | | 0.0030 | | | | 2.39 (1.73 - 3.30) | | | <.0001 | | |  |
| Not impaired (ref) |  |  |  |  |  |  |  |  |  |  |  |  |  |  |  |
| Organization |  | | | | | | | | | | | | | |  |
| Impaired | 0.94 (0.71 - 1.24) | 0.65 | 0.89 (0.68 - 1.17) | | 0.40 | | | | 1.16 (0.84 - 1.60) | | | 0.37 | | |  |
| Not impaired (ref) |  |  |  |  |  |  |  |  |  |  |  |  |  |  |  |
| Memory |  | | | | | | | | | | | | | |  |
| Impaired | 1.32 (1.05 - 1.66) | 0.018 | 1.32 (1.05 - 1.66) | | 0.016 | | | | 0.98 (0.74 - 1.30) | | | 0.88 | | |  |
| Not impaired (ref) |  |  |  |  |  |  |  |  |  |  |  |  |  |  |  |
| Distress |  | | | | | | | | | | | | | |  |
| Depression |  | | | | | | | | | | | | | |  |
| Yes | 1.05 (0.87 - 1.27) | 0.59 | 1.05 (0.88 - 1.27) | | 0.58 | | | | 0.90 (0.72 - 1.12) | | | | 0.33 | |  |
| No (ref) |  |  |  |  |  |  |  |  |  |  |  |  |  |  |  |
| Anxiety |  | | | | | | | | | | | | | |  |
| Yes | 1.89 (1.29 - 2.76) | 0.0010 | 2.01 (1.38 - 2.93) | | <.0001 | | | | 2.05 (1.35 - 3.12) | | | | <.0001 | |  |
| No (ref) |  |  |  |  |  |  |  |  |  |  |  |  |  |  |  |
| Somatization |  | | | | | | | | | | | | | |  |
| Yes | 1.95 (1.25 - 3.03) | 0.0030 | 1.41 (0.89 - 2.21) | | 0.14 | | | | 2.19 (1.32 - 3.63) | | | | 0.0020 | |  |
| No (ref) |  |  |  |  |  |  |  |  |  |  |  |  |  |  |  |
| Pain |  | | | | | | | | | | | | | |  |
| Yes | 1.01 (0.83 - 1.24) | 0.90 | 0.99 (0.81 - 1.22) | | 0.96 | | | | 0.95 (0.75 - 1.22) | | | | 0.70 | |  |
| No (red) |  |  |  |  |  |  |  |  |  |  |  |  |  |  |  |
| Emotion regulation* somatization | 0.37 (0.20 - 0.70) | 0.0021 | 0.55 (0.29 - 1.03) | | 0.062 | | | | 0.46 (0.23 - 0.93) | | | | 0.030 | |  |
| **Impaired Emotion Regulation with Somatization** | 0.54 (0.30 - 0.95) | 0.03 | 0.84 (0.47 - 1.50) | | | | 0.56 | | | 1.10 (0.57 - 2.12) | | | | 0.78 | |
| **Impaired Emotion Regulation without Somatization** | 1.44 (1.08 - 1.91) | 0.012 | 1.53 (1.16 - 2.03) | | | | 0.0030 | | | 2.39 (1.73 - 3.30) | | | | <.0001 | |

Analyses accounted for under-sampling of acute lymphoblastic leukemia (ALL) in the CCSS cohort using weighted polytomous logistic regression; analyses were also adjusted for cubic splines (five knots at 20, 25, 30, 35 and 45 years) of age at questionnaire. Models were adjusted for sociodemographic factors, treatment exposures, relapse, and secondary malignancy status. Ref=reference category. Reference category for alcohol use models: no use.

Supplemental Table 6 | Multivariable multinomial logistic regression model examining alcohol use among survivors of childhood cancer – with moderating effects of somatization on organization

| **Variables** | **Alcohol Use**  **(N=8245)** | | | | | |
| --- | --- | --- | --- | --- | --- | --- |
|  | **Occasional** | | **Risky** | | **Heavy** | |
|  | **OR (95% CI)** | ***P*** | **OR (95% CI)** | ***P*** | **OR (95% CI)** | ***P*** |
| Neurocognitive concerns |  |  |  |  |  |  |
| Task efficiency |  |  |  |  |  |  |
| Impaired | 0.67 (0.53 - 0.85) | <.0001 | 0.58 (0.46 - 0.73) | <.001 | 0.60 (0.45 - 0.81) | <.0001 |
| Not impaired | *Reference* | | | | | |
| Emotional regulation |  |  |  |  |  |  |
| Impaired | 1.21 (0.94 - 1.55) | 0.13 | 1.38 (1.08 - 1.77) | 0.010 | 2.09 (1.56 - 2.78) | <.0001 |
| Not impaired | *Reference* | | | | | |
| Organization |  |  |  |  |  |  |
| Impaired | 1.03 (0.76 - 1.39) | 0.86 | 0.86 (0.63 - 1.16) | 0.32 | 1.42 (1.00 - 2.01) | 0.051 |
| Not impaired | *Reference* | | | | | |
| Memory |  |  |  |  |  |  |
| Impaired | 1.32 (1.05 - 1.66) | 0.017 | 1.33 (1.06 - 1.66) | 0.015 | 0.97 (0.73 - 1.29) | 0.84 |
| Not impaired | *Reference* | | | | | |
| Distress |  |  |  |  |  |  |
| Depression |  |  |  |  |  |  |
| Yes | 1.06 (0.88 - 1.27) | 0.56 | 1.06 (0.88 - 1.27) | 0.60 | 0.89 (0.72 - 1.11) | 0.32 |
| No | *Reference* | | | | | |
| Anxiety |  |  |  |  |  |  |
| Yes | 1.81 (1.25 - 2.63) | 0.0020 | 1.95 (1.35 - 2.82) | <.001 | 2.00 (1.32 - 3.01) | <.001 |
| No | *Reference* | | | | | |
| Somatization |  |  |  |  |  |  |
| Yes | 1.48 (1.04 - 2.11) | 0.029 | 1.07 (0.75 - 1.54) | 0.71 | 2.00 (1.34 - 2.98) | <.001 |
| No | *Reference* | | | | | |
| Pain |  |  |  |  |  |  |
| Yes | 1.01 (0.83 - 1.24) | 0.91 | 0.99 (0.81 - 1.22) | 0.94 | 0.96 (0.75 - 1.22) | 0.72 |
| No | *Reference* | | | | | |
| Organization *  Somatization | 0.57 (0.29 - 1.10) | 0.094 | 1.06 (0.54 - 2.06) | 0.87 | 0.34 (0.15 - 0.73) | 0.0060 |
| **Impaired Organization**  **with Somatization** | 0.58 (0.32 - 1.07) | 0.081 | 0.90 (0.49 - 1.66) | 0.75 | 0.48 (0.23 - 0.98) | 0.043 |
| **Impaired Organization without Somatization** | 1.03 (0.76 - 1.39) | 0.86 | 0.86 (0.63 - 1.16) | 0.32 | 1.42 (1.00 - 2.01) | 0.051 |

Analyses accounted for under-sampling of acute lymphoblastic leukemia (ALL) in the CCSS cohort using weighted polytomous logistic regression; analyses were also adjusted for cubic splines (five knots at 20, 25, 30, 35 and 45 years) of age at questionnaire. Models were adjusted for sociodemographic factors, treatment exposures, relapse, and secondary malignancy status. Reference category for alcohol use models: no use.

Supplemental Table 7 | Multivariable multinomial logistic regression model examining alcohol use among survivors of childhood cancer – with moderating effects of pain on organization

| **Variables** | **Alcohol Use**  **(N=8245)** | | | | | |
| --- | --- | --- | --- | --- | --- | --- |
|  | **Occasional** | | **Risky** | | **Heavy** | |
|  | **OR (95% CI)** | ***P*** | **OR (95% CI)** | ***P*** | **OR (95% CI)** | ***P*** |
| Neurocognitive concerns |  |  |  |  |  |  |
| Task efficiency |  |  |  |  |  |  |
| Impaired | 0.68 (0.54 - 0.86) | <0.0001 | 0.59 (0.47 - 0.74) | <.001 | 0.61 (0.46 - 0.81) | <.0001 |
| Not impaired | *Reference* | | | | | |
| Emotional regulation |  |  |  |  |  |  |
| Impaired | 1.19 (0.94 - 1.53) | 0.15 | 1.37 (1.08 - 1.75) | 0.011 | 2.07 (1.56 - 2.76) | <.0001 |
| Not impaired | *Reference* | | | | | |
| Organization |  |  |  |  |  |  |
| Impaired | 0.77 (0.56 - 1.07) | 0.12 | 0.69 (0.50 - 0.94) | 0.020 | 1.08 (0.75 - 1.56) | 0.69 |
| Not impaired | *Reference* | | | | | |
| Memory |  |  |  |  |  |  |
| Impaired | 1.32 (1.05 - 1.65) | 0.017 | 1.31 (1.05 - 1.64) | 0.017 | 0.97 (0.73 - 1.29) | 0.85 |
| Not impaired | *Reference* | | | | | |
| Distress |  |  |  |  |  |  |
| Depression |  |  |  |  |  |  |
| Yes | 1.06 (0.88 - 1.28) | 0.53 | 1.06 (0.88 - 1.28) | 0.53 | 0.90 (0.72 - 1.12) | 0.35 |
| No | *Reference* | | | | | |
| Anxiety |  |  |  |  |  |  |
| Yes | 1.77 (1.22 - 2.57) | 0.0030 | 1.90 (1.31 - 2.74) | <.001 | 1.96 (1.30 - 2.95) | 0.001 |
| No | *Reference* | | | | | |
| Somatization |  |  |  |  |  |  |
| Yes | 1.26 (0.93 - 1.71) | 0.13 | 1.05 (0.77 - 1.42) | 0.77 | 1.54 (1.09 - 2.19) | 0.015 |
| No | *Reference* | | | | | |
| Pain |  |  |  |  |  |  |
| Yes | 0.94 (0.76 - 1.16) | 0.55 | 0.90 (0.72 - 1.11) | 0.33 | 0.93 (0.72 - 1.20) | 0.59 |
| No | *Reference* | | | | | |
| Organization*Pain | 1.81 (1.03 - 3.19) | 0.039 | 2.18 (1.24 - 3.85) | 0.0070 | 1.25 (0.64 - 2.41) | 0.51 |
| **Impaired Organization**  **with Pain** | 1.40 (0.86 - 2.28) | 0.17 | 1.50 (0.92 - 2.44) | 0.10 | 1.34 (0.75 - 2.39) | 0.32 |
| **Impaired Organization**  **without Pain** | 0.77 (0.56 - 1.07) | 0.12 | 0.69 (0.50 - 0.94) | 0.020 | 1.08 (0.75 - 1.56) | 0.69 |

Analyses accounted for under-sampling of acute lymphoblastic leukemia (ALL) in CCSS cohort using weighted polytomous logistic regression; analyses were also adjusted for cubic splines (five knots at 20, 25, 30, 35 and 45 years) of age at questionnaire. Models were adjusted for sociodemographic factors, treatment exposures, relapse, and secondary malignancy status. Reference category for alcohol use models: no use.

Supplemental Table 8| Multivariable multinomial logistic regression model examining alcohol use among survivors of childhood cancer – with moderating effects of somatization on memory

| **Variables** | **Alcohol Use**  **(N=8245)** | | | | | |
| --- | --- | --- | --- | --- | --- | --- |
|  | **Occasional** | |  | | **Heavy** | |
|  | **OR (95% CI)** | ***P*** | **OR (95% CI)** | ***P*** | **OR (95% CI)** | ***P*** |
| Neurocognitive concerns |  |  |  |  |  |  |
| Task efficiency |  |  |  |  |  |  |
| Impaired | 0.67 (0.53 - 0.85) | <.0001 | 0.58 (0.46 - 0.73) | <.001 | 0.60 (0.45 - 0.81) | <.0001 |
| Not impaired | *Reference* | | | | | |
| Emotional regulation |  |  | |  |  |  |
| Impaired | 1.21 (0.94 - 1.55) | 0.14 | 1.38 (1.08 - 1.77) | 0.0090 | 2.10 (1.57 - 2.80) | <.0001 |
| Not impaired | *Reference* | | | | | |
| Organization |  |  |  |  |  |  |
| Impaired | 0.93 (0.70 - 1.22) | 0.59 | 0.88 (0.67 - 1.16) | 0.36 | 1.15 (0.83 - 1.59) | 0.40 |
| Not impaired | *Reference* | | | | | |
| Memory |  |  |  |  |  |  |
| Impaired | 1.37 (1.07 - 1.74) | 0.011 | 1.38 (1.08 - 1.75) | 0.0090 | 1.11 (0.83 - 1.50) | 0.48 |
| Not impaired | *Reference* | | | | | |
| Distress |  |  |  |  |  |  |
| Depression |  |  |  |  |  |  |
| Yes | 1.06 (0.88 - 1.28) | 0.55 | 1.06 (0.88 - 1.27) | 0.57 | 0.89 (0.72 - 1.11) | 0.32 |
| No | *Reference* | | | | | |
| Anxiety |  |  |  |  |  |  |
| Yes | 1.83 (1.25 - 2.66) | 0.0020 | 1.97 (1.36 - 2.86) | <.0001 | 2.02 (1.33 - 3.05) | <.0001 |
| No | *Reference* | | | | | |
| Somatization |  |  |  |  |  |  |
| Yes | 1.48 (0.96 - 2.28) | 0.072 | 1.28 (0.83 - 1.98) | 0.27 | 2.20 (1.38 - 3.52) | <.0001 |
| No | *Reference* | | | | | |
| Pain |  |  |  |  |  |  |
| Yes | 1.01 (0.82 - 1.24) | 0.92 | 0.99 (0.81 - 1.22) | 0.93 | 0.95 (0.74 - 1.21) | 0.68 |
| No | *Reference* | | | | | |
| Memory*Somatization | 0.75 (0.41 - 1.36) | 0.34 | 0.70 (0.38 - 1.27) | 0.24 | 0.45 (0.23 - 0.88) | 0.021 |
| **Impaired Memory**  **with Somatization** | 1.02 (0.58 - 1.81) | 0.94 | 0.96 (0.54 - 1.71) | 0.89 | 0.50 (0.26 - 0.96) | 0.038 |
| **Impaired Memory**  **without Somatization** | 1.37 (1.07 - 1.74) | 0.011 | 1.38 (1.08 - 1.75) | 0.0090 | 1.11 (0.83 - 1.50) | 0.48 |

Analyses accounted for under-sampling of acute lymphoblastic leukemia (ALL) in the CCSS cohort using weighted polytomous logistic regression; analyses were also adjusted for cubic splines (five knots at 20, 25, 30, 35 and 45 years) of age at questionnaire. Models were adjusted for sociodemographic factors, treatment exposures, relapse, and secondary malignancy status. Reference category for alcohol use models: no use.
