# Supplementary material for: Neurocognitive impairment and substance use in adult survivors of childhood cancer: a cross-sectional analysis from the Childhood Cancer Survivor Study
Source: eClinicalMedicine. 2026 Apr 29;95:103924. doi: 10.1016/j.eclinm.2026.103924 (PMC13141803; doi:10.1016/j.eclinm.2026.103924)
Supplement: Supplementary Fig. S1 [file mmc2.docx]

## Supplemental Figure 1. Consort diagram

Questionnaire not completed by self-report **N = 1102**

Age less than 18 years old at assessment **N = 27**

Survivors who completed Follow-up questionnaires

**N = 14,247**

Adult survivors who self-completed Follow-up questionnaires with complete NCQ

**N = 11151**

Any missing NCQ domains **N = 1967**

Adult survivors who completed Follow-up questionnaires with complete NCQ

**N = 12253**

Adult survivors who completed Follow-up questionnaires

**N = 14220**
